# Changed adipocytokine concentrations in colorectal tumor patients and morbidly obese patients compared to healthy controls

**DOI:** 10.1186/1471-2407-12-545

**Published:** 2012-11-23

**Authors:** Andreas Hillenbrand, Juliane Fassler, Nadine Huber, Pengfei Xu, Doris Henne-Bruns, Markus Templin, Hubert Schrezenmeier, Anna Maria Wolf, Uwe Knippschild

**Affiliations:** 1 Department of General and Visceral Surgery, University Hospital of Ulm, Albert-Einstein-Allee 23, 89081, Ulm, Germany; 2 NMI at the University of Tuebingen, Markwiesenstr. 55, 72770, Reutlingen, Germany; 3 Institute of Clinical Tranfusion Medicine and Immunogenetics, Helmholtzstr. 10, 89081, Ulm, Germany

**Keywords:** Adipokine, Adipocytokine, Cytokine, Colorectal cancer, Morbid obesity, Adiponectin, Leptin, Resistin

## Abstract

**Background:**

Obesity has been associated with increased incidence of colorectal cancer. Adipose tissue dysfunction accompanied with alterations in the release of adipocytokines has been proposed to contribute to cancer pathogenesis and progression. The aim of this study was to analyze plasma concentrations of several adipose tissue expressed hormones in colorectal cancer patients (CRC) and morbidly obese (MO) patients and to compare these concentrations to clinicopathological parameters.

**Methods:**

Plasma concentrations of adiponectin, resistin, leptin, active plasminogen activator inhibitor (PAI)-1, monocyte chemotactic protein (MCP)-1, interleukin (IL)-1 alpha, and tumor necrosis factor (TNF)-alpha were determined in 67 patients operated on for CRC (31 rectal cancers, 36 colon cancers), 37 patients operated on for morbid obesity and 60 healthy blood donors (BD).

**Results:**

Compared to BD, leptin concentrations were lowered in CRC patients whereas those of MO patients were elevated. Adiponectin concentrations were only lowered in MO patients. Concentrations of MCP-1, PAI-1, and IL-1 alpha were elevated in both CRC and MO patients, while resistin and TNF-alpha were similarly expressed in MO and CRC patients compared to BD. Resistin concentrations positively correlated with tumor staging (p<0.002) and grading (p=0.015) of rectal tumor patients.

**Conclusions:**

The results suggest that both MO and CRC have low-grade inflammation as part of their etiology.

## Background

Obesity is an increasing health problem not only for industrialized countries but also for most other parts of the world affecting all ages [1]. Besides the established complications like cardiovascular disorders and type II diabetes mellitus, obesity has not only been associated with a 1.5–3.5-fold increased cancer incidence, but also with increased cancer mortality, especially in morbidly obese patients (BMI > 40 kg/m^2^) [2,3]. A positive association between obesity and risks for cancers like endometrial or kidney cancer is well documented [4-6]. There is also an association between obesity and colorectal cancer, however, this association appears to be stronger in males, particularly with visceral adiposity and weaker and less consistent in women, underlining gender-specific differences regarding the correlation of obesity and cancer development [7-10].

A number of mechanisms have been proposed for the adverse effect of obesity on colorectal cancer risk including the distribution of body fat, alteration in hormonal patterns, obesity-related inflammation, and metabolic disturbances [11]. White adipose tissue has been increasingly recognized as an important endocrine organ. The physiological functions of adipose tissue are changed in obesity, leading to an altered secretion of adipocytokines, which may influence cancer pathogenesis and progression [12,13]. These adipocytokines, particularly adiponectin, leptin, tumor necrosis factor-alpha (TNF-α), and some proinflammatory interleukins like interleukin 1α (IL-1α) [14] may indicate an association between obesity and colorectal cancer by influencing the obesity associated low grade inflammation and the growth and proliferation of tumor stroma and malignant cells within [15-17]. Furthermore, interaction between tumor and stromal cells may influence tumor progression. Tumor associated macrophages, which are major components of stroma and attracted by MCP-1, have been reported to play a role in tumor progression [18].

Knowledge of the pathophysiological mechanisms of these various protein signals underlying the association between obesity and cancer origin may be important for the development of preventive and therapeutic strategies for malignancies. These proteins involved in various signaling pathways are called adipocytokines. Hereafter, the term adipokine refers to the adipose tissue expressed hormones leptin, resistin and adiponectin and the term cytokines refers to IL-1α and TNF-α. Monocyte chemotactic protein-1 (MCP-1) is a chemokine, a protein that acts as a chemical messenger, and active PAI-1 belongs to the family of serine protease inhibitors. In literature there are contradictory reports regarding adipokine concentrations in colorectal cancer patients [19]. Furthermore, recent studies suggest that changes in the expression of adipose tissue expressed hormones may reflect a mechanism linking obesity to tumor genesis [20,21].

The aim of this study is, (i) to provide an adipokine profile in three different groups (colorectal cancer patients, morbidly obese patients and healthy blood donors), (ii) to assess the influence of altered adipocytokine expression on tumorigenesis, (iii) to investigate the association between plasma adipokine concentrations and clinicopathological characteristics of CRC and (iv) to demonstrate similarities in the cytokine/chemokine profile of CRC and MO patients. Our data indicate that MO and CRC have both chronic low-grade inflammation as part of their etiology.

## Methods

### Patients

The patients were divided into three subgroups: Subgroup 1: patients with colorectal cancer (CRC); subgroup 2: morbidly obese (MO) patients; subgroup 3: healthy blood donors (BD) as control group. Inclusion criteria to participate voluntarily in the study were age >18 years, no further malignant or rapidly progressing or hematologic underlying disease, no HIV/AIDS, or cytotoxic therapy given within the previous 6 months. The study was performed with the permission of the independent local ethics committee of the University of Ulm (approvals 112/2003 and 73/2009). An informed consent of all MO and CRC patients was obtained prior to surgery in the author’s hospital.

Fasting blood samples from CRC and MO patients were collected preoperatively at 7 AM. Samples from BD were taken at various times in a non-fasting state (10 ml venous blood, collected in a chilled syringe with EDTA). All samples were frozen in liquid nitrogen and stored in the tissue bank of the Department of General and Visceral Surgery of the University Hospital of Ulm, Germany.

### Cytokine and adipocyte expressed hormone measurement and reagents

Multiplex analysis kits for IL-1α, TNF-α and Linco-Kit human serum adipokine (panel A, panel B) for MCP-1, active PAI-1, leptin, resistin and adiponectin were obtained from Millipore, Hamburg, Germany. In brief, the appropriate cytokine standards and samples, diluted in plasma dilution buffer, were added to wells of a filtered plate. The samples were incubated with 50 μl of the antibody-coupled microsphere set on a plate shaker in the dark at room temperature for 30 min. The samples were washed three times with 100 μl wash buffer. Freshly diluted secondary detection antibody (25 μl/well) was added to the wells and then incubated on a plate shaker in the dark at room temperature. Thereafter, samples were washed three times with 100 μl wash buffer. Fifty microliters of strepavidin-PE (16 μg/ml in assay buffer) was added to each well, and incubation continued on a plate shaker at room temperature for the first 10 min. Unbound analytes were filtered through the wells using the vacuum manifold. The bound beads were washed three times with 100 μl wash buffer. After the last wash step, 125 μl of assay buffer was added to each well and the plate was placed on a plate shaker set at 500 rpm (G-force: 0.84 g) for 1 min and then for 3 min at a reduced speed of 300 rpm (G-force: 0.30 g). Finally, samples were analyzed on the Luminex system in accordance with the manufacturer’s instruction at the NMI of the University of Tübingen. The lowest traceable value is according to the sensitivity of the method 0.3 ng/ml for leptin and 9.2 pg/ml for IL-1α. Intra-assay precision (Intra-CV%) and inter-assay precision (Inter-CV%) are provided in Table 1.

**Table 1:** **Median and range of adiponectin, resistin, leptin, active PAI-1, MCP-1, IL-1α and TNF-**α **plasma concentrations of patients operated on for colorectal carcinoma (CRC) and morbid obesity (MO) as well as healthy blood donors (BD)**

**Parameters Intra-CV% Inter-CV%**	**CRC (n=67)**	**MO (n=37)**	**BD (n=60)**	**CRC vs. BD**	**MO vs. BD**	**CRC vs. MO**
Adiponectin (μg/ml)	17.1	**↓**9.4	14.7	n.s.	p<0.001	p<0.001
3.9 / 7.8	(4.3 – 51.0)	(3.8 – 57.8)	(5.7 – 54.0)			
Adiponectin females (μg/ml)	21.9	**↓**12.4	22.5	n.s.	p<0.001	p=0.002
	(7.0 – 40.5)	(5.3 – 57.8)	(9.8 – 54.0)			
Adiponectin males (μg/ml)	14.2	**↓**8.3	11.8	n.s.	p=0.001	p<0.001
	(4.3 – 51.0)	(3.8 – 12.3)	(5.7 – 30.7)			
Leptin (ng/ml)	**↓**2.6	**↑**45.0	10.9	p<0.001	p<0.001	p<0.001
6.4 / 9.6	(0.3 – 33.3)	(1.7 – 97.5)	(0.3 – 58.5)			
Leptin females	**↓**6.7	**↑**47.3	15.8	p=0.011	p<0.001	p<0.001
	(0.3 - 33.3)	(16.1 – 97.5)	(0.7 – 58.5)			
Leptin males	**↓**1.7	**↑**34.2	4.7	p=0.001	p<0.001	p<0.001
	(0.3 – 19.6)	(1.7 – 90.9)	(0.3 – 19.1)			
Resistin (ng/ml)	10.9	9.9	10.8	n.s.	n.s.	n.s.
8.3 / 11.1	(4.2 – 43.5)	( 3.7 – 83.6)	(5.2 – 24.9)			
MCP-1 (pg/ml)	**↑**127	**↑**108	43.9	p<0.001	p<0.001	p=0.047
5.1 / 11.2	(22.7 – 451)	(39.6 – 293)	(12.1 – 150)			
Active PAI-1 (ng/ml)	**↑**13.2	**↑**57.5	6.0	p<0.001	p<0.001	p<0.001
17.4 / 20.0	(2.4 – 53.2)	(3.7 – 176.7)	( 2.4 – 31.3)			
TNF-α(pg/ml)	4.8 (n=63)	4.0	3.9	n.s.	n.s.	n.s.
9.4 / 10.7	(2.4 – 74.7)	(2.4 – 26.8)	( 2.4 – 10.9)			
IL-1α (pg/ml)	**↑**52.4 (n=63)	**↑**59.4	11.0	p<0.001	p<0.001	n.s.
20.0 / 35.7	(9.2 – 1322)	(9.2 – 210.0)	( 9.2 – 168)			

**↓↑:** Significantly elevated/lowered concentrations compared to BD.The inter- and intra-assay CVs are given below the corresponding parameters.

### Statistical analysis

All values were expressed as median with range. Statistical analysis was performed using WinSTAT software (Version 2009.1; R. FitchSoftware). Data were statistically analyzed using the Mann–Whitney tests, and correlations between the different adipocytokines by the non-parametic Spearman’s correlation test. The correlation coefficient is indicated by r. Statistical significance was declared at p<0.05 and a tendency at 0.05<p<0.10. No adjustments were made for multiple statistical comparisons.

## Results

### Baseline characteristics of the study participants

Morbidly obese patients (24 female, 13 male) had a median age of 45 years (range: 17 – 59) and a median BMI of 52.0 kg/m^2^ (range: 33.5 – 78.0). The healthy BD (30 female, 30 male) had a median age of 45 years (range: 19 – 71 years). Body mass index (BMI) was not ascertained in this group. The CRC subgroup (25 female, 42 male) included patients with colon cancer (36) and rectal cancer (31). Median age of CRC patients was 66 years (range: 28 – 91 years), median BMI was 27.1 kg/m^2^ (range: 18.9 – 38.3). Rectal cancer was staged as stage I in 11, stage II in 3, stage III in 11, and stage IV in 6 patients. Colon cancer was staged as stage I in 8, stage II in 12, stage III in 12, and stage IV in 4 patients [22]. The BMIs of MO patients were significantly higher compared to those of CRC patients (p<0.01; WMU-T-Test). Gender-specific anthropometric data of all three groups are listed in Table 2.

**Table 2:** Gender-specific anthropometric data (age and BMI) of patients with colorectal cancer (CRC), morbid obesity (MO) and healthy blood donors (BD)

	**Age [years] (median; range)**	**BMI [kg/m**^**2**^**] (median; range)**
CRC (n=67)	66.0 years (28 – 91)	27.1 kg/m^2^ (18.9 – 38.3)
Female patients (n=25)	66.0 years (28 – 91)	26.4 kg/m^2^ (18.9 – 35.5)
Male patients (n=42)	65.5 years (37 – 90)	27.2 kg/m^2^ (20.6 – 38.3)
Female rectal tumor patients (n=12)	64.5 years (41 – 84)	27.6 kg/m^2^ (18.9 – 35.5)
Male rectal tumor patients (n=19)	64.0 years (37 – 90)	27.2 kg/m^2^ (20.6 – 38.3)
Female colon tumor patients (n=13)	66.0 years (28 – 91)	26.3 kg/m^2^ (22.0 – 32.2)
Male colon tumor patients (n=23)	67.0 years (46 – 90)	26.8 kg/m^2^ (21.6 – 36.3)
MO (n=37)	45.0 years (17 – 59)	52.0 kg/m^2^ (33.5 – 78.0)
Female patients (n=24)	46.5 years (23 – 59)	52.4 kg/m^2^ (35.0 – 78.0)
Male patients (n=13)	42.0 years (17 – 59)	49.1 kg/m^2^ (33.5 – 70.4)
BD (n=60)	45.0 years (19 – 71)	Not ascertained
Female control (n=30)	44.0 years (19 – 60)	Not ascertained
Male control (n=30)	46.0 years (19 – 71)	Not ascertained

Adipocytokine concentrations were clearly changed in CRC and MO patients compared to healthy BD. For a better overview, plasma concentrations of adiponectin, resistin, leptin, active PAI-1, MCP-1, IL-1α, and TNF-α and corresponding significant changes are shown in Table 1. A gender-specific difference was seen in the values of adiponectin and leptin. In females, adiponectin and leptin values were elevated compared to males. Resistin, active PAI-1, MCP-1, IL-1α, and TNF-αshowed no gender-specific differences.

### Adipokines (adiponectin, resistin and leptin)

There was no significant gender-specific difference in adiponectin concentrations in the CRC group compared to the BD group, but, as expected, adiponectin was lowered in the MO group (p<0.001 females and males). However, significant differences in the plasma leptin concentrations were detected in CRC patients compared to MO patients. Leptin concentrations were significantly lowered in CRC patients and significantly elevated in the MO compared to the BD group (p≤0.01; Table 1). Resistin concentrations showed no differences between the groups.

**Figure 1:**
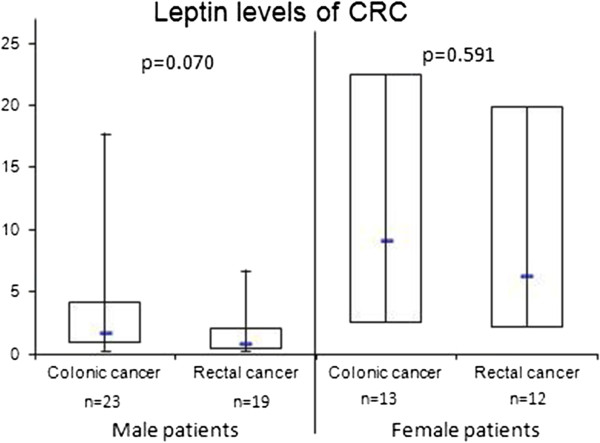
**Leptin concentrations of CRC patients according to tumor localization (colon cancer vs. rectal cancer).** Male colon cancer patients had (not significant) elevated leptin concentrations compared to male rectal cancer patients (median colon cancer leptin: 1.7 ng/ml; range: 0.3 – 19.6 ng/ml vs. median rectal cancer leptin: 0.8 ng/ml; range: 0.3 – 6.7 ng/ml; p=0.062). There was no difference in female patients (leptin: 9.2 ng/ml; range: 0.3 – 30.8 ng/ml vs. median rectal cancer leptin: 6.3 ng/ml; range: 0.3 – 33.3 ng/ml; p=0.07). The top and bottom of the rectangle represent the 25th and 75th percentile. The line within the rectangle represents the median. The whiskers extend from the 5th percentile to the 95th percentile.

Plasma leptin concentrations in male patients with colon carcinoma tended to be elevated compared to male patients with rectal carcinoma (median leptin: 1.7 ng/ml; range: 0.3 – 19.6 ng/ml vs. 0.9 ng/ml; range: 0.3 – 6.7 ng/ml; p=0.070). Females with colon/rectal cancer had comparable leptin concentrations (6.3 ng/ml vs. 9.2 ng/ml; p=0.591; Figure 1). Adiponectin concentrations in females and males showed no difference in rectal and colon cancer groups (female rectal: 22.5 μg/ml; female colon: 21.9 μg/ml; male rectal: 13.9 μg/ml; male colon 15.9 μg/ml). In all four subgroups the BMI was not significantly different (female colon/rectal: 26.4 kg/m^2^ vs. 27.6 kg/m^2^; male colon/rectal: 27.0 kg/m^2^ vs. 27.9 kg/m^2^). There was no significant difference in resistin concentrations among all patient groups.

### Correlation of adipokines in colon cancer and rectal cancer patients with tumor size based on T-staging and tumor grading

In patients suffering from rectal carcinoma, resistin correlates positively with tumor size based on T-staging [23] (r=0.49; p<0.01) and tumor grading (r=0.39; p=0.02). If focused solely on males, this trend was even stronger (correlation of resistin with T-staging in males: r=0.70; p<0.01; correlation of resistin with tumor grading in males: r=0.56; p<0.01). This effect was not seen in the female subgroup (correlation of resistin with T-staging in females: r=0.07; p=0.41; correlation of resistin with tumor grading in females: r=0.13; p=0.34). However, after multivariate logistic regression with adjustment for age and gender there was no significant correlation (Table 3). Possibly the loss of significance could be due to the limited size of subgroups. In patients suffering from colon cancer, there was no correlation of resistin concentrations with tumor size based on T-staging and tumor grading. Plasma adiponectin and leptin concentrations showed no correlation with tumor size or tumor grading in colon or rectal cancer.

**Table 3:** Correlation of resistin concentrations in rectal tumor patients with tumor size based on T-staging and tumor grading – multivariate logistic regression with adjustment for age and gender

**Variable**	**Parameter**	**p-value**	**95%-Confidence interval (lower limit)**	**95%-Confidence interval (upper limit)**
Intercept	2.24	0.59	−6.28	10.76
Age	0.09	0.13	−0.03	0.22
Gender	1.93	0.22	−1.24	5.10
Grade	1.06	0.67	−4.04	6.15
Stage	2.11	0.19	−1.09	5.31

### *Monocyte chemotactic protein-1, active PAI-1, TNF-*α*, IL-1*α

Monocyte chemotactic protein-1, active PAI-1, and IL-1α were significantly elevated in CRC and MO patients compared to BD controls whereas no significant changes in TNF-αwere detected. However, it is striking that the plasma concentrations in both MO and CRC patients are changed in the same direction. Tumor necrosis factor-α(TNF-α) and IL-1αplasma concentrations are comparable in MO and CRC patients. In contrast, active PAI-1 was higher in MO than CRC patients, and MCP-1 was higher in CRC than MO patients.

## Discussion

The aim of this study was to demonstrate similarities in the cytokine/chemokine profile of colorectal cancer patients and morbidly obese since obesity is linked to an increased risk of cancer [24] possibly due to adipose tissue dysfunction accompanied with alterations in the release of adipocytokines. We found increased circulating concentrations of MCP-1, PAI-1, and IL-1αin both CRC and MO patients, while resistin and TNF-α were similarly expressed in MO and CRC patients. Adiponectin concentrations were lowered only in MO patients, whereas leptin concentrations were lowered in CRC patients.

Adipokines, in particular leptin and adiponectin, have attracted much attention because of their potential role in the development and progression of various obesity-related malignancies [25,26]. We found lowered leptin concentrations in CRC patients in agreement with other studies [27]. Sălăgeanu and co-workers even report a negative correlation between leptin and T-staging [19]. Interestingly, we found a significant difference in leptin concentrations of male patients with colon carcinoma vs. patients with rectal carcinoma. This difference was not seen in female patients. Since both patient groups have approximately the same BMI, this difference cannot be explained by an obesity-related leptin elevation alone. A similar association of colon cancer and elevated leptin concentrations in males was observed by Stattin and co-workers [28]. Gender-specific concentrations were significantly lowered in patients with CRC compared to the control group. As suspected, leptin concentrations showed a positive correlation to patients’ BMI.

Adiponectin may be a molecular marker for obesity and CRC but evidence from large prospective studies is limited [29]. Adiponectin is decreased in the obese and is supposed to exert an anti-inflammatory and anti-cancerous activity. Its absence is related to several obesity-related malignancies including colorectal cancer [30]. In several further studies, low plasma adiponectin concentrations were an independent risk factor for colorectal cancer and its precursory adenoma [31-33]. However, we report, as do others [34,35], no association between plasma adiponectin concentration and CRC. The association between adiponectin concentrations and the risk of colorectal cancer still remains controversial, especially as there are different fractions of adiponectin (total/high molecular weight/non-high molecular weight adiponectin) with different hypothesized biological activities [29].

Resistin, a protein with proinflammatory properties, is mainly expressed from macrophages rather than adipocytes [36]. We observed similar resistin concentrations in both CRC and MO. Resistin correlates positively with tumor size based on T-staging and tumor grading. However, no significant results after adjustment for age and gender could be observed by multivariate logistic regression due to the small number of cases. Further studies found resistin being significantly elevated in cancer patients and in several studies stage progression significantly correlates with resistin concentrations [37,38]. We found this trend to be stronger in males and only in rectal cancer and not in colon cancer. In most other studies, there is neither a gender specific differentiation nor one between colon and rectal cancer [27,38].

Tumor necrosis factor-α(TNF-α) is a key cytokine, not secreted by adipocytes but produced by both macrophages and malignant cells [39,40]. Expression and secretion of TNF-α increase with obesity and correlate positively with body mass index [41]. Several studies suggest that TNF-α is involved in obesity-related insulin resistance and that TNF-α is one of the most important mediators of inflammation [42]. We found a slight but not significant elevation of TNF-α in CRC patients compared to MO and BD.

Plasminogen activator inhibitor-1 (PAI-1), a physiological inhibitor of urokinase-type and tissue-type plasminogen activator, is an integral part of the fibrinolytic system and contributes to the process of tumor invasion and metastasis due to degrading of the cellular matrix [43,44]. It was shown that plasma PAI-1 concentrations correlated with metastatic disease in CRC patients [45] and with the outcome of rectal cancer [46]. We also found significantly elevated PAI-1 concentrations in CRC patients as well as in MO compared to BD as control group.

Monocyte chemotactic protein-1 (MCP-1) is a chemokine that accumulates and influences tumor associated macrophages. This accumulation of macrophages seems to have an impact on cancer pathogenesis by influencing either growth promotion or growth inhibition depending upon the particular setting [47]. The presence of the infiltrating macrophages has been shown to correlate with cancer metastasis and poor prognosis in a variety of human carcinomas [48]. The expression of MCP-1 has been reported in several tumors including human colorectal cancer [49,50]. We found elevated concentrations in MO as well as in CRC patients.

Interleukin-1 is a major mediator of inflammation and is known to be up-regulated in many tumor types and has been implicated as a factor in tumor progression [51]. Interleukin-1 consists of two molecular species, IL-1α and IL-1β, both of which exert similar biological functions through the IL-1 type I receptor [52]. As in cancer, elevated IL-1 concentrations are reported in obesity [17]. Since macrophages accumulate in both tissues, in adipose tissue in MO and cancer tissue in CRC patients, elevated IL-1α concentrations are unsurprising in both our groups.

Examining these cytokines, it is striking that plasma concentrations of MO and CRC are changed in the same direction. The amplitude of change of MO and CRC to BD is comparable in TNF-α and IL-1α plasma concentrations. Active PAI-1 is proportionally more elevated in MO compared to CRC. Monocyte chemotactic protein-1, however, is proportionally more elevated in CRC compared to MO. This could be explained by the fact that PAI-1 is not only produced by the visceral adipose tissue (increased in the morbidly obese) but also by steatotic liver tissue in morbidly obese patients [53,54]. Monocyte chemotactic protein-1 is produced by cancer cells and multiple different host cells within the tumor microenvironment and serves as one of the key mediators of interactions between tumor and host cells [55]. Similar cytokine/chemokines/PAI-1 profile in MO and CRC patients reflects the obesity and tumor associated chronic inflammation. However, the specific influence of adipokines on tumor development and progression is still not fully understood and has to be addressed.

Our study has several strengths and limitations. All patients were recruited and operated on in the same hospital. Blood samples of all three groups were collected via the same protocol, were simultaneously stored, and processed with the same assay. Patients of the MO group were not only overweight, but truly morbidly obese (median BMI of MO patients: 52.0 kg/m^2^). In the absence of standard values for many adipocytokines we provide a profile for the three different groups.

The main limitation of our study is the missing BMI of the BD group due to the fact that in general body weight and height are not routinely measured in the Institute of Clinical Transfusion Medicine and Immunogenetics. This obviously affects the interpretation of the results. Further limitations are the small sample size and the associated lack of significance in statistical analysis of the data and the lack of correlation of resistin levels with tumor size based on T-staging and tumor grading in patients suffering from colon cancer. Further, no information was collected regarding a screening colonoscopy for colorectal cancer in the BD group.

## Conclusion

The aim of this study was to demonstrate similarities in the cytokine/chemokine profile of colorectal cancer patients and morbidly obese since obesity is linked to an increased risk of cancer, possibly due to adipose tissue dysfunction. We demonstrated similarities in the cytokine/chemokine profile of CRC and MO patients. We found significantly lowered leptin concentrations (gender dependent) in the carcinoma group compared to BD. In male patients with colon carcinoma, we report elevated leptin concentrations compared to leptin concentrations in rectal tumor patients. Further, we found a positive correlation of resistin and tumor size based on T-staging in CRC males. Similarities in the cytokine/chemokine profile of colorectal cancer patients and the morbidly obese indicate that both have chronic low-grade inflammation as part of their etiology. Future studies are warranted to confirm these results and to elucidate the underlying mechanisms.

## Abbreviations

BD: Blood donors; CRC: Colorectal carcinoma; CV%: Coefficient of variation; IL-1: Interleukin-1; MCP-1: Monocyte chemotactic protein-1; MO: Morbidly obese patients; PAI-1: Active plasminogen activator inhibitor-1; TNF-α: Tumor necrosis factor (TNF)-α.

## Competing interests

All authors declare that they have no competing interests.

## Authors’ contributions

All the authors have contributed substantially to the submitted work and have read and revised the paper and approved the final version. In particular AH, UK and AMW participated in the design of the study, data acquisition, analysis and drafting of the manuscript. JF, NH and PX analyzed the data and provided the literature research. AH, MT and UK analyzed the plasma levels. DHB initialized the work, provided laboratory results and gave approval for submission. HS provided data of blood donors. AMW and UK obtained ethical approval.

## Authors’ information

Anna Maria Wolf and Uwe Knippschild shared senior authorship.

## Pre-publication history

The pre-publication history for this paper can be accessed here:

http://www.biomedcentral.com/1471-2407/12/545/prepub
